# Interregional outbreak of *Salmonella Typhimurium* linked to fresh cheese: A case–case study guided by whole-genome sequencing (WGS), Portugal, March–June 2024

**DOI:** 10.1017/S0950268826101538

**Published:** 2026-05-04

**Authors:** Sebastian von Schreeb, Leonor Silveira, Ângela Pista, João Paulo Gomes, Mónica Oleastro, Sara Gomes Raposo, Valentyna Lutsiv, Juan Rachadell, Helder Pinto, Patrícia Correia Rico, Luísa Pestana, Vera Leal Pessoa, António Carlos Silva, Ana Dinis, Maria Helena Almeida, Kostas Danis, Paula Vasconcelos, Pedro Pinto Leite, Vasco Ricoca Peixoto

**Affiliations:** 1 Public Health Emergencies Operations Centre (CESP), Direcção-Geral da Saúde, Portugal; 2 Directorate of Information and Analysis (DSIA), Direcção-Geral da Saúde, Portugal; 3 ECDC Fellowship Programme, Field Epidemiology path (EPIET), European Centre for Disease Prevention and Control, Sweden; 4 Department of Infectious Diseases, National Reference Laboratory for Gastrointestinal Infections, National Institute of Health Doctor Ricardo Jorge, Portugal; 5 Public Health Unit, Arrábida Local Health Unit, Portugal; 6 Public Health Unit, Central Alentejo Local Health Unit, Portugal; 7 Regional Public Health Department for Lisbon and Tagus Valley, Direcção-Geral da Saúde, Portugal; 8 NOVA National School of Public Health, Public Health Research Centre, Comprehensive Health Research Center, CHRC, REAL, CCAL, NOVA University Lisbon, Lisbon, Portugal

**Keywords:** Outbreak, Salmonella, Case-case study, Whole Genome sequencing, Food and Water Borne Diseases

## Abstract

In 2024, an outbreak of *Salmonella typhimurium* affected two regions in Portugal. To identify the vehicle, we conducted a case–case study using a ‘same disease, different time period’ design. We compared *S. typhimurium* cases linked by whole-genome sequencing (WGS) (cluster cases) with salmonellosis cases notified in 2023 (historical cases) and calculated odds ratios (OR) for food exposures in surveillance data using logistic regression. We performed WGS on 58 isolates from the outbreak period (11/03/2024–2118/06/2024), and all belonged to a single cgMLST cluster (HierCC HC5_410,410). Compared with the 552 historical cases, cluster cases more frequently reported fresh cheese consumption (OR 18; 95% CI: 8.5–38). We visited the implicated cheese production site, identified food safety non-conformities, and enforced hygiene measures. Environmental and product specimens collected at the visit tested negative for Salmonella spp. Taken together, the most plausible vehicle in this outbreak was fresh cheese. The case–case design enabled a rapid, low-cost analysis to support targeted investigation using surveillance data. Using WGS cluster cases as the case definition, rather than all *S. typhimurium* cases during the outbreak period, yielded a higher OR in the case–case study, increasing confidence in the findings. We recommend this combined approach as part of the toolkit for foodborne outbreak investigations in Portugal in similar contexts.

## Introduction

*Salmonella enterica* subsp. *enterica* serovar Typhimurium is a zoonotic pathogen and a common cause of gastroenteritis in humans [1]. It is most often contracted by eating contaminated food or via direct contact with infected animals or their environment. Outbreaks are commonly associated with food products, especially poultry, eggs, pork products, and fresh produce. In Portugal, *S. typhimurium* is the second most common serotype, after *Salmonella Enteritidis*, with yearly numbers of cases ranging between 262 and 535 since 2015, far below the EU/EEA average [2].

On 30 April 2024, the Directorate-General of Health of Portugal (DGS) was informed through the epidemic intelligence network about an unusual increase in a *S. typhimurium* strain by the National Reference Laboratory (NRL) for Gastrointestinal Infections at Instituto Nacional de Saúde Doutor Ricardo Jorge (INSA), representing more than a fivefold rise compared with the same period in the previous year. The increase had started around 25 March, with samples arriving from the regions of Alentejo and Lisbon and Tejo Valley (LVT), which are located adjacent in the centre of the country.

Concurrently, clinicians at the hospital in Évora, in Alentejo, had noticed an increase in salmonellosis cases among patients admitted with gastrointestinal complaints, and the Local Public Health Units of Alentejo and Arrábida observed an increase in the number of cases reported through the National Epidemiological Surveillance System (SINAVE) that includes clinical and laboratory notifications. The epidemiologic inquiries conducted by the Arrábida local public health unit indicated that many cases had reported consuming a specific cheese from a local producer.

An outbreak investigation team was set up, which included stakeholders from local public health units, regional public health units, NRL/INSA, the Directorate-General of Food and Veterinary (DGAV), and DGS. The investigation aimed to estimate the magnitude and describe the characteristics of the outbreak, identify the source, and promote public health measures to prevent further spread, as described in this outbreak report.

## Methods

### Laboratory methods

The NRL/INSA receives isolates on a volunteer basis from several public and private hospitals, and two of the major private laboratories in Portugal. All isolates received in the outbreak period underwent routine serotyping (Kauffman–White–Le Minor scheme) [3], antimicrobial susceptibility testing (AST), following recommendations from the European Committee on Antimicrobial Susceptibility Testing (EUCAST) [4], and were subjected to whole-genome sequencing (WGS) [5]. Raw reads were subjected to EnteroBase for the generation of high-quality reads and cluster analysis [6–8].

### Data collection

Surveillance data were extracted from SINAVE, which includes clinical and laboratory notifications of salmonellosis, in line with EU case definitions [9]. Following a clinical notification, public health physicians at local public health units routinely contact cases by telephone to complete a standard questionnaire (epidemiological investigation form). These include food items considered at risk for salmonellosis consumed during the incubation period, including unpasteurized milk, fresh cheese, ice cream, eggs, cream, mayonnaise, shellfish, raw or undercooked meats, raw vegetables (not washed or peeled), and raw fruit (not washed or peeled).

Between 31 May and 4 June, we re-interviewed outbreak cases by telephone using a follow-up questionnaire to supplement data from the case notifications. This questionnaire complemented information on specific high-risk foods, including cheese, milk, and eggs, on consumption frequency, purchase locations, and specific brands. It also covered additional potential exposures. Furthermore, we conducted telephone interviews with fresh cheese producers mentioned in the free-text responses of the notification forms, using a semi-structured interview guide. The interview guide covered questions about product details, any recent staff illnesses, supplier relationships, and customer interactions.

### Case–case study

We conducted a retrospective case–case study comparing historical cases to cluster cases, corresponding to the ‘same disease, different time period’ design described previously [10]. We defined historical cases as salmonellosis cases reported in Portugal between 1 January and 31 December 2023. We defined cluster cases as *S. typhimurium* cases reported during the outbreak period (11 March–18 June 2024) that belonged to the genetic cluster STm410410 (HC5 410,410), as determined by WGS. For robustness, we repeated the comparison using two broader case definitions: all salmonellosis cases reported during the outbreak period (regardless of serotype), and *S. typhimurium* cases reported during the outbreak period.

We described the outbreak by time, place, and person, and calculated odds ratios (OR) using univariable and multivariable logistic regression for the exposures reported in the salmonellosis epidemiological investigation form, adjusting for sex, age, and other exposures. To account for potential seasonal patterns in food consumption, we repeated the analysis using historical cases restricted to the same period in the previous year (11 March–18 June 2023).

#### Food and environmental investigation

In Portugal, local public health units initiate investigations when they detect unusual increases in reported cases, and the regional and national levels become involved when events extend beyond a single jurisdiction. On 17 April, Arrábida local health unit observed a sudden rise in salmonellosis cases with repeated references to the same producer and therefore conducted a targeted food and environmental investigation by visiting a producer repeatedly mentioned in the notification forms to collect specimens and assess compliance with hygiene measures. A second visit was made to a marketplace where the products from this producer were sold, which was also repeatedly mentioned, for specimen collection. All specimens were tested at the NRL for the occurrence of *Salmonella* spp. using the Vitek immunodiagnostic assay system easy Salmonella (VIDAS ESLM) method with ISO 6579-1:2017 confirmation [11]. On 7 June, a follow-up visit to the local cheese producer was conducted with two representatives from the Directorate-General for Food and Veterinary Affairs (DGAV) to verify compliance with previously requested food safety and hygiene procedures.

Telephone interviews with cheese producers in the Alentejo region were conducted. These interviews included questions on types of products produced, distribution practices, ingredient suppliers, and any reports of illness among staff or customers. We did not perform a formal traceback investigation.

## Results

**Figure 1.**
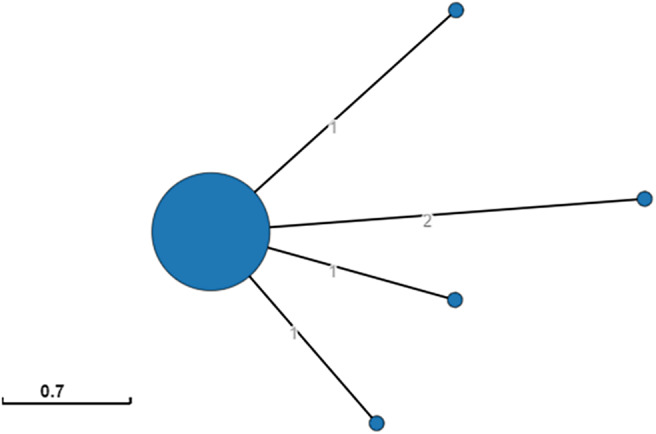
Minimum spanning tree of the STm410410 cluster, generated with the MSTreeV2 method of GrapeTree based on allelic diversity found among 65 human isolates. The size of the filled circles is proportional to the number of isolates it represents. The numbers on the connecting lines represent the allele differences between isolates.

Overall, 65 isolates were sent to the reference laboratory and included in the genomic analysis. All isolates belonged to serovar *S. typhimurium* ST19. A minimum spanning tree generated in EnteroBase (cgMLST V2 + HierCC V1) indicated genetic proximity among all the 65 isolates, belonging to HC5 410,410 and sharing 0–2 allelic differences (Figure 1).

### Case–case study: Descriptives

**Figure 2.**
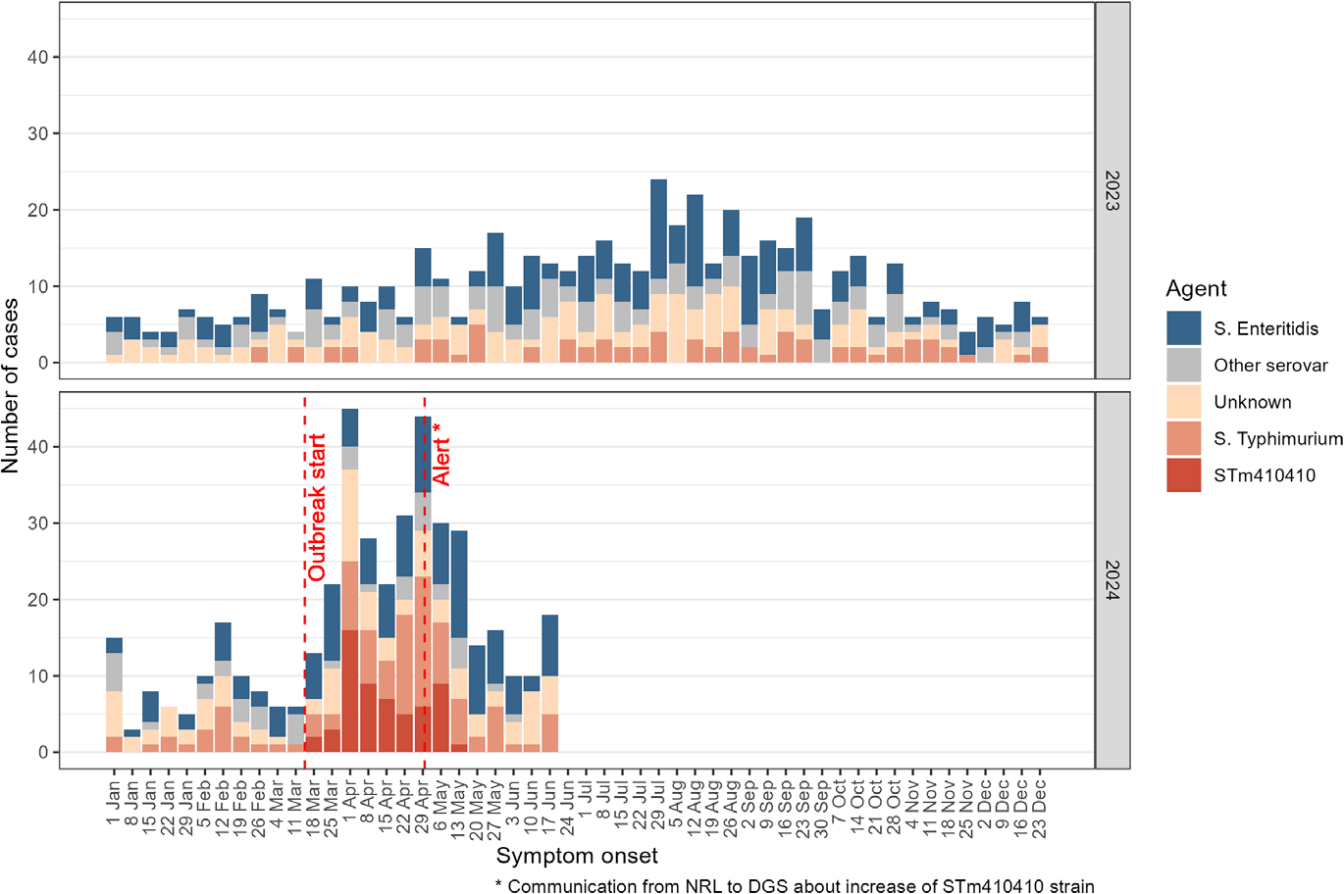
Salmonellosis cases in Portugal by causative agent and week of symptom onset, Portugal, Jan 1st, 2023, to June 18th, 2024. The outbreak was defined to start on March 11th, 2024. Cases with agent STm410410 were all caused by S. Typhimurium and belong to the same cluster

A total of 326 salmonellosis cases were notified through SINAVE with symptom onset between 11 March and 18 June 2024 (Figure 2). Among these, 140 (43%) were reported as *S. typhimurium*, 102 (31%) as *S. enteritidis*, 25 (8%) as other serovars, and 59 (18%) as *Salmonella* spp. unknown serovar. Among the 65 isolates identified as part of the STm410410 cluster by WGS, 58 could be linked to the SINAVE case data and were included in the epidemiological analysis.

**Figure 3.**
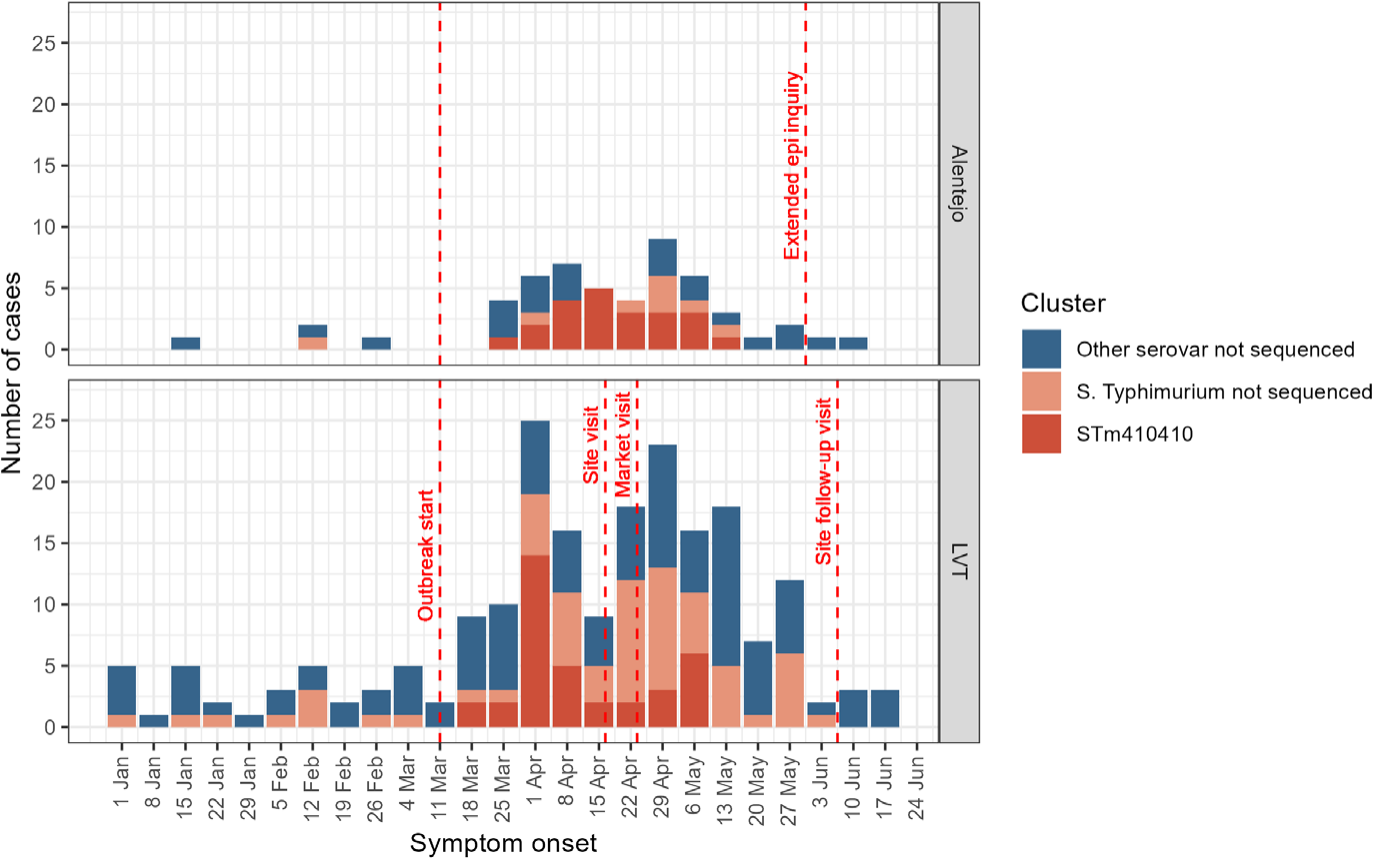
Number of cases of salmonellosis in the regions Alentejo and LVT by week of symptom onset, Portugal, Jan 1st to Jun 11th, 2024. Cases belonging to the STm410410 cluster of Salmonella Typhimurium are shown in red. The LVT region has roughly eight times the population size of the Alentejo region

**Figure 4.**
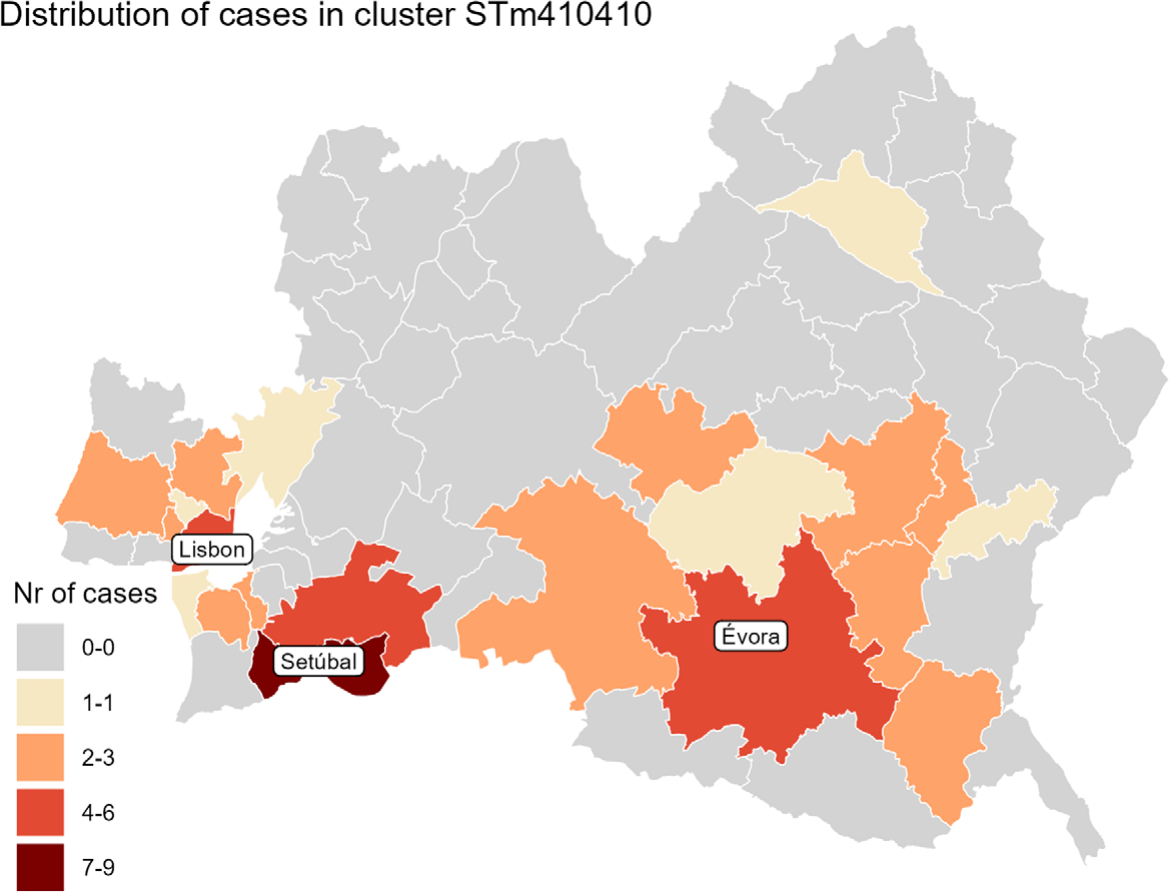
Geographical distribution of Salmonella Typhimurium STm410410, Lisbon and Tejo Valley region (LVT) and Alentejo region, Portugal, 2024.

The outbreak began on 11 March, peaked on 1 April, and ended on 18 June 2024. Cases belonging to the STm410410 cluster were spreading concurrently in the LVT and Alentejo region, with a larger peak in the number of cases observed in LVT in early April 2024 (Figure 3). The geographical distribution of cases was centred on the municipalities of Setúbal, Évora, and Lisboa, with cases dispersed from the Atlantic coast towards the Spanish border, spanning a distance of around 150 km (Figure 4).

### Case–case study: Comparison of exposure

Overall, 58 STm410410 cluster cases and 552 historical cases were included in the case–case study (Table 1). The cluster cases had a higher percentage of people in the age group 65+ compared to historical cases (40% vs. 20%, OR 3.1, 95% CI: 1.6–5.8) in the univariable analysis, while no significant association was found when adjusting for food exposures and age (adjusted OR 1.69, 95% CI: 0.71–3.9). Cluster cases were only reported from the region of LVT (61%) and Alentejo (39%), while historical cases were reported from all regions in Portugal (Table 1).

**Table 1.** Comparison of *Salmonella typhimurium* STm410410 cluster cases to historical cases (baseline)

Characteristic	Category	Descriptives		Univariable analysis			Multivariable analysis		
		Baseline *N* = 552^a^	STm410410 *N* = 58^a^	OR	95% CI	*p*-value	aOR	95% CI	*p*-value
Sex	Female	247 (45%)	25 (43%)	ref.	–		–	–	
	Male	305 (55%)	33 (57%)	1.1	0.62, 1.7	0.8	1.02	0.48, 2.20	>0.9
Age (years)	0–17	343 (62%)	24 (41%)	ref.	–		–	–	
	18–64	97 (18%)	11 (19%)	1.6	0.74, 3.4	0.2	0.97	0.32, 2.67	>0.9
	65+	112 (20%)	23 (40%)	2.9	1.6, 5.4	<0.001	1.69	0.71, 3.89	0.2
Region	Alentejo	18 (3.3%)	22 (38%)						
	Açores	3 (0.5%)	0 (0%)						
	Algarve	18 (3.3%)	0 (0%)						
	Centro	87 (16%)	0 (0%)						
	LVT	210 (38%)	36 (62%)						
	Norte	205 (37%)	0 (0%)						
	Madeira	11 (2.0%)	0 (0%)						
Exposure	Unpasteurized milk	3 (0.7%)	2 (4.2%)	5.7	0.76, 36	0.056	1.04	0.09, 11.6	>0.9
	Fresh cheese	17 (4.2%)	21 (44%)	17	8.5, 38	<0.001	12.7	5.07, 33.0	<0.001
	Ice cream	47 (12%)	3 (6.3%)	0.51	0.12, 1.5	0.3	0.35	0.06, 1.40	0.2
	Eggs	204 (48%)	35 (70%)	2.5	1.4, 4.8	0.005	1.86	0.83, 4.21	0.13
	Cream	24 (6.0%)	3 (6.3%)	1.0	0.24, 3.1	>0.9	1.08	0.15, 4.56	>0.9
	Mayonnaise	31 (7.7%)	3 (6.4%)	0.82	0.19, 2.4	0.7	0.72	0.11, 3.30	0.7
	Shellfish	8 (2.0%)	6 (13%)	7.1	2.2, 21	<0.001	2.32	0.41, 12.0	0.3
	Undercooked meats	50 (12%)	2 (4.2%)	0.31	0.05, 1.0	0.11	0.42	0.05, 1.95	0.3
	Raw vegetables	62 (16%)	15 (32%)	2.6	1.3, 4.9	0.006	1.02	0.31, 3.22	>0.9
	Raw fruit	84 (21%)	19 (39%)	2.4	1.3, 4.4	0.007	1.70	0.58, 4.74	0.3

a *n* (%).

Abbreviations: CI, confidence interval; OR, odds ratio. Historical cases were defined as cases of salmonellosis notified in Portugal in 2023. In the multivariable analysis, variables were adjusted for exposures, sex, and age.

Compared with historical cases, cluster cases were more likely to have consumed fresh cheese (OR 18, 95% CI = 8.5–38), shellfish (OR 7.1, 95% CI = 2.2–21), unpasteurized milk (OR 5.9, 95% CI = 0.76–36), and raw fruit (OR 2.4, 95% CI = 1.3–4.4) in the univariable analysis. In the multivariable analysis, cluster cases were associated with fresh cheese consumption (adjusted OR 12.7, 95% CI 5.1–33), while the other exposures showed no significant association after adjustments.

When we compared historical cases with *S. typhimurium* outbreak cases and salmonellosis outbreak cases, the OR for fresh cheese decreased to 5.6 (95% CI: 3.0–11) and 3.5 (95% CI: 1.9–6.6), respectively (Table 2).

**Table 2.** Comparison of historical cases (baseline) to outbreak cases using three different outbreak case definitions

Characteristic	Baseline *N* = 552^a^	Outbreak salmonellosis *N* = 326^a^	OR	95% CI	*p*-value	Outbreak Typhimurium *N* = 140^a^	OR	95% CI	*p*-value	STm410410 *N* = 58^a^	OR	95% CI	*p*-value
Unpasteurized milk	3 (0.7%)	5 (2.0%)	2.78	0.68, 13.7	0.2	3 (2.9%)	4.00	0.73, 21.9	0.093	2 (4.2%)	5.86	0.76, 36.2	0.056
Fresh cheese	17 (4.2%)	33 (13%)	3.52	1.94, 6.61	<0.001	28 (27%)	8.45	4.45, 16.5	<0.001	21 (44%)	17.8	8.50, 38.3	<0.001
Ice cream	47 (12%)	27 (11%)	0.94	0.56, 1.55	0.8	8 (7.8%)	0.65	0.28, 1.34	0.3	3 (6.3%)	0.51	0.12, 1.47	0.3
Eggs	204 (48%)	150 (59%)	1.56	1.14, 2.14	0.006	71 (66%)	2.05	1.33, 3.21	0.001	35 (70%)	2.49	1.35, 4.83	0.005
Cream	24 (6.0%)	20 (8.2%)	1.41	0.75, 2.60	0.3	7 (6.9%)	1.17	0.45, 2.66	0.7	3 (6.3%)	1.04	0.24, 3.14	>0.9
Mayonnaise	31 (7.7%)	27 (11%)	1.50	0.87, 2.59	0.14	7 (6.9%)	0.88	0.35, 1.96	0.8	3 (6.4%)	0.82	0.19, 2.41	0.7
Shellfish	8 (2.0%)	16 (6.5%)	3.43	1.49, 8.59	0.005	12 (12%)	6.44	2.59, 16.9	<0.001	6 (13%)	7.05	2.23, 21.3	<0.001
Undercooked meats	50 (12%)	26 (11%)	0.83	0.49, 1.36	0.5	8 (7.7%)	0.59	0.25, 1.21	0.2	2 (4.2%)	0.31	0.05, 1.03	0.11
Raw vegetables	62 (16%)	47 (19%)	1.30	0.86, 1.98	0.2	24 (24%)	1.67	0.97, 2.82	0.058	15 (32%)	2.55	1.27, 4.91	0.006
Raw fruit	84 (21%)	70 (29%)	1.51	1.04, 2.18	0.028	34 (33%)	1.88	1.16, 3.01	0.010	19 (39%)	2.38	1.26, 4.40	0.007

a *n* (%).

Abbreviations: CI, confidence interval; OR, odds ratio. Historical cases are defined as salmonellosis cases notified in Portugal in 2023. Outbreak cases are defined as: (1) all notified salmonellosis cases during the outbreak period, (2) *S. typhimurium* cases during the outbreak period, and (3) STm410410 cluster cases confirmed by WGS during the outbreak period.

We performed a univariable sensitivity analysis restricting historical cases to the regions affected in the outbreak (Supplementary Material 1). We also performed a univariable sensitivity analysis restricting historical cases to those occurring during the same calendar period as the outbreak in the previous year (11 March–18 June 2023) (Supplementary Material 2). Both sensitivity analyses found associations for the same main items as in the main univariable analysis, but with wider confidence intervals. Because of fewer cases and missing data on food exposures in these restricted datasets, multivariable analyses were not performed.

### Follow-up questionnaire and producer interviews

Among the 22 WGS-linked outbreak cases in the Alentejo region, 13 responded to a follow-up telephone interview. Of those, three (23%) reported exposure to fresh cheese and nine (69%) reported exposure to eggs and milk prior to symptom onset, which were bought in different locations or from their own production. Eight (62%) reported having bought pet food containing chicken or meat, all from different producers.

Of the four fresh cheese producers in Alentejo contacted due to being mentioned in the notification forms, two responded; none had any distribution link to the specific producer in Arrábida, nor reported any disease among their staff or customers. Therefore, no targeted food or environmental investigation was conducted in the Alentejo region. The remaining two did not respond to repeated attempts to contact them by telephone, and no further contact was made.

### Outbreak control measures

In the Arrábida public health unit, among the first 17 notified *Salmonella* cases, eight (47%) reported consumption of a specific brand of fresh cheese from the same producer during the incubation period; of these, seven reported purchasing it at the same market. During the visit to the producer, food safety measures were reinforced, including review and strengthening of hygiene and sanitation procedures, verification of cleaning and disinfection protocols, assessment of staff food handling practices, and reinforcement of traceability and record-keeping requirements. Three cheese specimens and two surface swabs were collected. At the visit to the market, four additional cheese specimens and three surface swabs were collected. All (100%) tested negative for *Salmonella* spp. During the follow-up visit on 7 June, an employee reported having had gastrointestinal symptoms after consuming seafood at a family gathering on 31 March, with symptoms appearing on 1 April and resolving without medical assistance. Only a limited number of the previously required food safety measures had been implemented, leading the Health Authority and DGAV to impose additional control measures. Due to delays in implementing these measures, production was suspended on 19 July until all requirements were met.

## Discussion

The case–case study suggested fresh cheese as the most plausible vehicle of the *S. typhimurium* outbreak, with the strength of association increasing when the case definition was more narrowly defined. A production site was visited, and hygiene measures were reinforced, although food and environmental samples tested negative.

This study is the first in Portugal to integrate WGS with the case–case study design in foodborne outbreak investigation. WGS is increasingly being used to investigate foodborne outbreaks internationally [12–14]. The case–case design, which compares outbreak cases with historical cases, has previously been recommended as an underutilized tool in outbreak investigation to reduce selection bias, recall bias, and costs while increasing timeliness, thereby supplementing a classic case–control study [10]. We found that integrating WGS to guide the case–case study enhanced the specificity of exposure-outcome associations.

The univariable analysis also identified weaker associations, with lower ORs and a smaller number of cases affected, and the second-highest estimate after fresh cheese was shellfish. In the multivariable analysis, only fresh cheese was associated with being a cluster case, suggesting that the association with shellfish was confounded by fresh cheese consumption. Furthermore, given the rarity of *S. typhimurium* in seafood [15, 16], we do not consider shellfish a plausible alternative explanation for the current outbreak.

This study has several limitations. First, due to a lack of data, we included historical cases from all of Portugal as comparison cases. This introduces a risk of representativeness bias due to geographic differences in exposure. To address this, we conducted two sensitivity analyses: first, restricting the historical cases to only include the affected regions, and second, to only include cases occurring during the same calendar period as the outbreak in the previous year. None of the sensitivity analyses indicated a representativeness bias in our main analysis, and both identified fresh cheese as the most probable source. Nevertheless, readers should be aware that, in case–case studies using a ‘same disease, different time period’ design, comparison cases should come from the same geographic population as the cases to minimize bias. Strengths of this design include reduced selection bias and recall bias due to the use of the same surveillance system, while limitations include the possibility that exposure patterns, such as dietary habits, may change over time. A comprehensive discussion of the strengths and limitations of this design compared to other case–case study designs is provided elsewhere [10]. A limitation of case–case studies compared to case–control studies is that historical cases do not accurately represent the background population because they are cases. For common exposures to *Salmonella* that are highly prevalent among historical cases, such as eggs, the design may underestimate the associated risk of exposure. A limitation in the current study is that isolate submissions to the NRL for WGS depended on voluntary participation by local laboratories and hospitals, which introduces a potential selection bias among serotyped and WGS-linked cases. It is possible that some Typhimurium and unknown serovar cases were actually cluster cases but were not sent to the NRL. We mapped the 15 entities reporting isolates to the NRL, which showed that they were mainly located in Lisbon, Setúbal, and Évora. However, inconsistent reporting formats prevented a formal sensitivity analysis. Finally, the follow-up questionnaire did not yield valuable information, possibly due to a recall bias among cases, as these follow-up interviews were conducted 3 weeks after the last symptom onset. Future investigations should aim to reduce this limitation by conducting interviews closer to the time of illness.

Portugal currently lacks specific guidelines to standardize the use of WGS in foodborne outbreak investigations, and existing general guidelines have not been updated since 2001 [17]. Considering that the local public health unit identified a potential outbreak and initiated a site visit, which was later supported by the findings from the WGS-informed case–case study, future revisions should clarify procedures for timely signal detection and investigation of outbreaks, including through WGS, in line with recommendations from the European Commission [18, 19]. This should include reviewing routine epidemiologic questionnaires, registries for routine surveillance, developing templates for trawling questionnaires, and guidance on descriptive and analytical epidemiology (including early case–case studies informed by WGS). Additionally, protocols for WGS sample selection, procedures after signal detection, and integration with SINAVE surveillance data are important to strengthen preparedness. To improve the timeliness of alerts, indicator-based surveillance using SINAVE and NRL laboratory data should implement procedures for signal detection by comparing case counts to expected cases, considering baselines at national, regional, and local levels.

## Conclusion

The combined epidemiological, environmental, and laboratory investigations were essential to determine the cause of this *S. typhimurium* outbreak, in line with the One Health approach to foodborne outbreak investigations. The epidemiological findings indicated fresh cheese as the most plausible vehicle, despite food and surface specimens testing negative for Salmonella spp. The case–case design provided a rapid, low-cost tool that leveraged existing surveillance data. Using WGS for the outbreak case definition increased confidence in the case–case study findings. We recommend integrating this combined approach into the standard toolkit for foodborne outbreak investigations in Portugal.

## Supporting information

10.1017/S0950268826101538.sm001 Von Schreeb et al. supplementary material 1 Von Schreeb et al. supplementary material

10.1017/S0950268826101538.sm002 Von Schreeb et al. supplementary material 2 Von Schreeb et al. supplementary material

## Data Availability

Data are available on reasonable request to the authors. Restrictions may apply to ensure data anonymity and compliance with national legislation. The R code used for analysis is available upon request.

## Supplementary material

The supplementary material for this article can be found at http://doi.org/10.1017/S0950268826101538.

## Preprint

The manuscript has not been previously published in a preprint.
