# Supplementary material for: Interregional outbreak of Salmonella Typhimurium linked to fresh cheese: A case–case study guided by whole-genome sequencing (WGS), Portugal, March–June 2024
Source: Epidemiol Infect. 2026 May 4;154:e68. doi: 10.1017/S0950268826101538 (PMC13231226; doi:10.1017/S0950268826101538)
Supplement: Von Schreeb et al. supplementary material 1 — Von Schreeb et al. supplementary material [file S0950268826101538sup001.docx]

**Table 3.** Sensitivity analysis of Table 1, restricting the baseline to the two outbreak regions (Lisbon and Tejo Valley and Alentejo). STm410410 cluster cases were compared with historical salmonellosis cases from these regions. Historical cases were defined as all salmonellosis cases notified in 2023 in LVT and Alentejo.

| **Characteristic** | **Baseline**  **(restricted region)** N = 228^1^ | **STm410410**  N = 58^1^ | **OR** | **95% CI** | **p-value** |
| --- | --- | --- | --- | --- | --- |
| Unpasteurized milk | 2 (50%) | 2 (50%) | 3.15 | 0.37, 26.9 | 0.3 |
| Unknown | 81 | 10 |  |  |  |
| Fresh cheese | 8 (28%) | 21 (72%) | 13.5 | 5.62, 35.5 | **<0.001** |
| Unknown | 81 | 10 |  |  |  |
| Ice cream | 8 (73%) | 3 (27%) | 1.15 | 0.24, 4.17 | 0.8 |
| Unknown | 82 | 10 |  |  |  |
| Eggs | 72 (67%) | 35 (33%) | 2.66 | 1.36, 5.38 | **0.005** |
| Unknown | 74 | 8 |  |  |  |
| Cream | 9 (75%) | 3 (25%) | 1.01 | 0.22, 3.55 | >0.9 |
| Unknown | 83 | 10 |  |  |  |
| Mayonnaise | 14 (82%) | 3 (18%) | 0.64 | 0.14, 2.08 | 0.5 |
| Unknown | 82 | 11 |  |  |  |
| Shellfish | 3 (33%) | 6 (67%) | 6.86 | 1.73, 33.6 | **0.008** |
| Unknown | 81 | 10 |  |  |  |
| Undercooked meats | 20 (91%) | 2 (9.1%) | 0.28 | 0.04, 1.00 | 0.091 |
| Unknown | 81 | 10 |  |  |  |
| Raw vegetables | 12 (44%) | 15 (56%) | 5.20 | 2.23, 12.4 | **<0.001** |
| Unknown | 83 | 11 |  |  |  |
| Raw fruit | 21 (53%) | 19 (48%) | 3.74 | 1.79, 7.87 | **<0.001** |
| Unknown | 83 | 9 |  |  |  |
| ^1^n (%) | | | | | |
| Abbreviations: CI = Confidence Interval, OR = Odds Ratio | | | | | |
