# Supplementary material for: Interregional outbreak of Salmonella Typhimurium linked to fresh cheese: A case–case study guided by whole-genome sequencing (WGS), Portugal, March–June 2024
Source: Epidemiol Infect. 2026 May 4;154:e68. doi: 10.1017/S0950268826101538 (PMC13231226; doi:10.1017/S0950268826101538)
Supplement: Von Schreeb et al. supplementary material 2 — Von Schreeb et al. supplementary material [file S0950268826101538sup002.docx]

**Table 4.** Sensitivity analysis of Table 1. The historical baseline is restricted to cases occurring during the same calendar period as the outbreak in the previous year (11 March to 18 June 2023).

| **Characteristic** | **Baseline**  **(restricted time period)**  N = 142^1^ | **STm410410**  N = 58^1^ | **OR** | **95% CI** | **p-value** |
| --- | --- | --- | --- | --- | --- |
| Unpasteurized milk | 3 (60%) | 2 (40%) | 1.59 | 0.20, 9.92 | 0.6 |
| Unknown | 29 | 10 |  |  |  |
| Fresh cheese | 7 (25%) | 21 (75%) | 11.7 | 4.69, 32.3 | **<0.001** |
| Unknown | 30 | 10 |  |  |  |
| Ice cream | 12 (80%) | 3 (20%) | 0.56 | 0.12, 1.85 | 0.4 |
| Unknown | 30 | 10 |  |  |  |
| Eggs | 58 (62%) | 35 (38%) | 2.33 | 1.17, 4.83 | **0.019** |
| Unknown | 26 | 8 |  |  |  |
| Cream | 5 (63%) | 3 (38%) | 1.40 | 0.28, 5.95 | 0.7 |
| Unknown | 32 | 10 |  |  |  |
| Mayonnaise | 9 (75%) | 3 (25%) | 0.79 | 0.17, 2.79 | 0.7 |
| Unknown | 29 | 11 |  |  |  |
| Shellfish | 2 (25%) | 6 (75%) | 7.86 | 1.73, 55.1 | **0.014** |
| Unknown | 30 | 10 |  |  |  |
| Undercooked meats | 10 (83%) | 2 (17%) | 0.44 | 0.07, 1.75 | 0.3 |
| Unknown | 31 | 10 |  |  |  |
| Raw vegetables | 19 (56%) | 15 (44%) | 2.22 | 1.00, 4.89 | **0.047** |
| Unknown | 33 | 11 |  |  |  |
| Raw fruit | 27 (59%) | 19 (41%) | 1.95 | 0.94, 4.00 | 0.070 |
| Unknown | 32 | 9 |  |  |  |
| ^1^n (%) | | | | | |
| Abbreviations: CI = Confidence Interval, OR = Odds Ratio | | | | | |
